# An analysis of factors influencing the demand for community-based integrated health and social care in Southwestern China

**DOI:** 10.3389/fpubh.2025.1684187

**Published:** 2025-11-28

**Authors:** Hanzhi Zheng, Min Yang, Qin Zeng, Shujuan Liao, Ping Li

**Affiliations:** 1 Department of Nursing, West China Second University Hospital, Sichuan University/West China School of Nursing, Sichuan University, Chengdu, Sichuan, China; 2 Key Laboratory of Birth Defects and Related Diseases of Women and Children (Sichuan University), Ministry of Education, Chengdu, Sichuan, China; 3 The Second People’s Hospital, Chengdu, Sichuan, China; 4 Chengdu University of TCM, Chengdu, Sichuan, China

**Keywords:** Andersen Health Service Utilization Behavior Model, CB-IHSC, long-term care, older adults, demand

## Abstract

**Objective:**

This cross-sectional study investigates the demand for CB-IHSC among older adults in Chengdu and identifies the factors influencing this demand, thereby providing a reference for developing and optimizing such services.

**Methods:**

From August to December 2023, 511 eligible older adults residing in the central districts of Chengdu were selected through convenience sampling. Guided by the Andersen Health Service Utilization Behavior Model, the study categorized influencing factors into three dimensions: predisposing factors, enabling factors and need factors. Chi-square tests were first used to identify statistically significant variables within each dimension. These variables were then included in a binary logistic regression model to assess their association with service demand. Model calibration was assessed using the Hosmer–Lemeshow test.

**Results:**

Demand for CB-IHSC was high (87.08%). Independent predictors included number of children (OR = 2.12), living with spouse (vs alone, Ref; OR = 0.48), lower income (OR = 0.40), convenient community medical access (OR = 1.75), children’s support (OR = 3.71), lower self-care ability (OR = 0.18), poorer self-rated health, and higher awareness. Policies should improve accessibility, financing, and public awareness to increase uptake.

**Conclusion:**

To support the sustainable development of CB-IHSC, Chengdu should continue strengthening capacity building in this sector. Efforts should be directed toward establishing diversified financing and support mechanisms, improving the accessibility and convenience of service delivery, and enhancing public awareness and education regarding CB-IHSC.

## 1 Introduction

With the global trend of an aging population, China is facing increasingly severe demographic and healthcare challenges. According to the National Bureau of Statistics, by the end of 2024, over 310 million people in China will be aged 60 and above, representing 22% of the total population. Among them, approximately 78% suffer from at least one chronic disease (1). Despite the growing demand for care services for older adults, the availability remains inadequate, with only 406,000 care institutions and 7.99 million beds nationwide (1). This imbalance between demand and supply underscores the urgent need to develop efficient long-term care system in China.

In response, the Chinese government has implemented policies to develop long-term care services and foster a supportive environment for older adults. Since the release of the Opinions on Accelerating the Development of the Care-for-older-adults in 2013, various models have emerged, including integrated health and care institutions for older adults, community-based integrated health and social care (CB-IHSC), and home-based integrated care. However, while these models have proliferated, gaps remain in their accessibility, quality, and integration.

The developed countries have already established comprehensive care system for older adults, such as the U.S. Program of All-Inclusive Care for the Elder Adults (PACE), the UK’s community-based older adults care framework, and Japan’s Kaigo Hoken (Long-Term Care Insurance) system. However, China’s care-for-older-adults industry is still in its early stages, with limited empirical research on CB-IHSC. Existing studies are primarily theoretical, focusing on framework development and public awareness, particularly in large cities. For instance, a study by Liu Xiaochu et al. reported a 66.3% demand for CB-IHSC in Luzhou (2), whereas a survey in Guangzhou indicated a higher demand of 86.26% (3). In Chengdu, older residents mainly require health management, smart care, medical consultations, psychological care, and rehabilitation nursing (4).

Further research across other megacities in China, such as Nanchang, Changsha, and Guangzhou, has identified multiple factors influencing demand for CB-IHSC, including health status, family support, and community environment (5, 6). For example, a survey in Guangzhou found a strong correlation between demand and factors such as health conditions, number of children, and self-rated health (3). Similarly, studies in Changsha highlighted the importance of family support, income levels, and the availability of medical resources in determining the use of community-based integrated care services (6). These findings underscore the need to understand how similar mechanisms operate in Chengdu.

The Andersen Health Service Utilization Behavior Model, proposed by Ronald M. Anderson in 1968, provides a robust theoretical framework for analyzing healthcare-seeking behavior. It posits that service utilization is jointly influenced by three categories of factors: Predisposing Characteristics (e.g., age, education), Enabling Resources (e.g., accessibility, family support), and Need (e.g., self-rated health, disease burden). This model’s relevance for understanding the demand for CB-IHSC is well-supported, as existing studies indicate that such demand arises from a complex interaction of individual health conditions, socio-economic factors, and service availability (7–9). Furthermore, the model’s applicability in the Chinese context has been extensively demonstrated in long-term care research conducted in settings such as Shaanxi, Suzhou, and Beijing (10–14). While alternative frameworks exist (e.g., the WHO’s ICOPE model), the Andersen model’s comprehensive and behavioral-focused structure makes it the most appropriate choice for systematically investigating the factors influencing demand for integrated care in this study.

### 1.1 The case of Chengdu

Chengdu, located in the southwest of China and as a national pilot for CB-IHSC, faces rapid population aging. Despite establishing 28 CB-IHSC complexes, challenges such as limited service scope and low public awareness persists (15, 16). Most studies on Chengdu focus on demand descriptions but lack a unified theoretical framework to explain the underlying drivers.

This study aims to fill this gap by applying the Andersen Health Service Utilization Behavior Model to identify key factors driving CB-IHSC demand in Chengdu. The following hypotheses are proposed, based on the model and prior literature:

*H1* (Need): Poorer self-rated health and lower self-care ability are associated with higher demand for CB-IHSC.

*H2* (Enabling—Access): Greater convenience of community medical care would be associated with higher odds of demand.

*H3* (Enabling—Resources): Higher income is positively associated with demand for CB-IHSC.

*H4* (Enabling—Family): Support from children positively influences demand. The more children an individual has, the lower the demand for services.

*H5* (Predisposing—Co-residence): Compared with living alone, co-residence with a spouse/children would be associated with lower odds due to informal care.

*H6* (Awareness): Higher awareness of CB-IHSC would be associated with higher odds of demand.

These hypotheses guide the model specification and analysis in the following sections.

Terminology: We use the term Community-Based Integrated Health and Social Care (CB-IHSC) throughout. Terms such as “integrated medical and social care” are treated as synonyms and, after first mention, replaced by CB-IHSC.

## 2 Materials and methods

### 2.1 Study design and participants

A cross-sectional survey was conducted among older residents aged 60 years and above in the main urban areas of Chengdu. The inclusion criteria were as follows: individuals who had resided for at least 6 months in one of the following districts—Jinjiang, Qingyang, Wuhou, Chenghua, Jinniu, Gaoxin, or Tianfu New District; individuals who regularly visited a designated community health service center for medical care; those aged 60 or older; and individuals capable of clear and logical communication. Exclusion criteria included: individuals with communication or expression impairments; individuals who refused to participate in the survey; respondents who completed the questionnaire in less than 3 minutes; those who selected the same response option throughout the questionnaire; individuals whose answers were internally inconsistent or logically contradictory. A total of 530 questionnaires were distributed, with 511 valid responses retained (effective response rate: 96.41%).

### 2.2 Data collection

Data were collected from August to December 2023 using electronic questionnaires administered at various community health service centers and nearby residential areas. Trained research assistants approached potential participants in waiting areas or common spaces of these centers, as well as in public areas of residential communities. They introduced themselves and the study purpose, explaining that the survey aimed to understand the needs and preferences of the CB-IHSC. Participation was voluntary, anonymous, and could be withdrawn at any time. Upon obtaining informed consent from the older participants or their accompanying family members, data were completed either by self-administration or with interviewer assistance as needed. Each survey session lasted approximately 5–10 min.

### 2.3 Questionnaire and measures

The questionnaire was designed based on the Andersen Health Service Utilization Behavior Model, which categorizes influencing factors into three dimensions: predisposing characteristics, enabling resources, and need factors. The following key factors were assessed:

*Predisposing characteristics*: Demographic characteristics: Age, gender, marital status, education level; Social structure variables: Occupation, living arrangements and number of children; Health beliefs: Factors such as attitudes toward health care and health-seeking behaviors.

*Enabling resources*: Financial resources: Monthly income, pension, monthly medical expenses and insurance coverage; Social support: Support from children, caregivers, or other family members (life care and financial support); Access to services: The proximity of healthcare facilities and the availability of medical resources in the community (ease of accessing medical services).

*Need factors*: Self-care ability: the ability to perform activities of daily living independently; Chronic conditions: Number of chronic illnesses the individual is managing; Health status: Self-reported health condition (e.g., good, fair, or poor); Awareness of services: awareness of CB-IHSC and actual demand for such services.

*Outcome variable*: Demand for CB-IHSC.

Demand, as a key concept in this study, is defined as the actual need or desire for CB-IHSC. It was operationalized in the questionnaire by asking participants the following question: “Do you currently have a need for community-based integrated health and social care services?” The response options were “Yes” or “No.” This was captured as a binary variable: “1” for respondents expressing a need (demand) for the services, and “0” for those indicating no demand.

### 2.4 Statistical methods

Data were processed and analyzed using SPSS 22.0 software. After assigning values to variables, both univariate and multivariate analyses were conducted. Univariate analysis was conducted using chi-square tests to identify significant variables associated with service demand. Multivariate analysis was performed using binary logistic regression. The model fit was evaluated by the Hosmer–Lemeshow test. The dependent variable was demand for CB-IHSC, coded as a binary variable: “0” representing no demand and “1” representing demand. In the binary logistic regression model, the relationship between the independent variables and the log-odds of demand is modeled. Let P represent the model-estimated probability that an individual has a demand for the services (i.e., Prob(Y = 1)). The odds of demand is then defined as P / (1 − P). The odds ratio (OR) for each independent variable, as reported in the results, represents the factor by which the odds of demand multiply for a one-unit change in the predictor variable, while holding other variables constant. An OR greater than 1 indicates that the predictor variable is associated with an increased likelihood of demand for the services, while an OR less than 1 suggests a decreased likelihood. Ordered categorical variables (e.g., income, satisfaction, self-rated health, convenience) were treated as ordinal predictors in the regression; dichotomous variables were binary-coded; nominal multi-level variables used the reference categories specified in Table 1 and Appendix Table A1.

**Table 1:** Analysis of factors influencing the demand for CB-IHSC among older participants with different characteristics.

Blocks	Independent variable	Category	*N*	Demand for the services		*p*	Chi-square
				No	Yes		
Predisposing characteristics	Education (Ref: Junior high and below)	Junior high school and below	261	20	241	*p*<0.001	18.625
		Bachelor degree	74	8	66		
		Junior college	59	12	47		
		Senior school	100	22	78		
		Master degree and above	17	4	13		
	Number of children (Ref: 0)	0	98	24	74	*p*<0.001	17.044
		1	161	16	145		
		2	133	18	115		
		≥3	119	8	111		
	Living arrangement (Ref: Alone)	Alone	80	9	71	*p*<0.001	33.561
		Nursing institution or other	7	6	1		
		Living with spouse	274	32	242		
		Living with children	150	19	131		
	Pre-retirement occupation (Ref: Public institution personnel)	Public institution personnel	100	15	85	*p* = 0.020	11.687
		Farmer	163	10	153		
		Other	72	10	62		
		Enterprise staff	104	16	88		
		no job	72	15	57		
Enabling resources	Income per month (RMB) (Ref: <3,000)	3,000–5,000	149	26	123	*p*<0.001	17.533
		<3,000	273	20	253		
		>5,000	89	20	69		
	Acceptable cost of medicine (RMB) (Ref:<1,000)	1,000–2000	109	16	93	*p*<0.001	21.454
		<1,000	352	34	318		
		2000–3,000	33	12	21		
		≥3,000	17	4	13		
	Convenience of community medical care (Ref: Inconvenience)	Inconvenience	48	11	37	*p* = 0.015	8.385
		Convenience	284	27	257		
		General	179	28	151		
	Source of care at the time of consultation/hospitalization (Ref: None)	Partner care only	98	17	81	*p* = 0.032	10.558
		One other source of care only	20	0	20		
		Child and child’s spouse care only	201	25	176		
		Two or more other sources of care	123	10	113		
		None	69	14	55		
	Sources of financial help in case of medical consultation/hospitalization (Ref: None)	Partner financial help only	70	13	57	*p* = 0.003	16.040
		One other kind of financial help only	29	8	21		
		Children and children’s spouses’ help only	186	17	169		
		Two and more kinds of other financial help	145	12	133		
		None	81	16	65		
	Children’s support (Ref: Do not support)	Do not support	101	19	82	*p* = 0.049	3.891
		Support	410	47	363		
Need factors	Self-care ability (Ref: No self-care ability)	Need help from others	104	34	70	*p*<0.001	45.739
		No self-care ability	6	0	6		
		Have self-care ability	401	32	369		
	Self-rated health (Ref: poor)	Poor	52	13	39	*p*<0.001	12.102
		Good	149	10	139		
		Fair	310	43	267		
	Awareness of the CB-IHSC (Ref: Never heard)	Know well	28	8	20	*p* = 0.003	11.331
		Never heard	200	16	184		
		Heard but not understand	283	42	241		

Pearson chi-square tests are computed within each variable using the No/Yes counts. Ref = reference category used in regression. Degrees of freedom (df) by variable — Education: df = 4; Number of children: df = 3; Living arrangement: df = 3; Pre-retirement occupation: df = 4; Income per month (RMB): df = 2; Acceptable cost of medicine(RMB): df = 3; Convenience of community medical care: df = 2; Source of care at the time of consultation/hospitalization: df = 4; Sources of financial help in case of medical consultation/hospitalization: df = 4; children’s support: df = 1; Self-care ability: df = 2; Self-rated health: df = 2; Awareness of the CB-IHSC: df = 2; Satisfaction with the CB-IHSC: df = 4. Ref as labeled; order does not imply reference status.

The hierarchical modeling approach was applied to assess how much additional variance in demand was explained by adding successive sets of factors. Three logistic regression models were constructed accordingly: Model 1 included statistically significant variables from the predisposing characteristics. Model 2 included significant variables from both predisposing and enabling factors. Model 3 included significant variables from all three dimensions: predisposing, enabling, and needs. Each model followed the general form. Changes in Cox & Snell R^2^ and Nagelkerke R^2^ were used to evaluate improvements in model fit, indicating how each block of variables contributed to explaining demand.


$$\text{Model}\;1:\text{logit}(p)=\alpha_{1}+\beta_{1}X_{\text{predisposing characteristics}}+\varepsilon_{1}$$

$$\begin{array}{l} \text{Model}\;2:\text{logit}(p)=\alpha_{2}+\beta_{21}X_{\text{predisposing characteristics}}+\\ \beta_{22}X_{\text{enabling resources}}+\varepsilon_{2} \end{array}$$

$$\begin{array}{l} \text{Model}\;3:\text{logit}(p)=\alpha_{3}+\beta_{31}X_{\text{predisposing characteristics}}+\\ \beta_{32}X_{\text{enabling resources}}+\beta_{33}X_{\text{needs}}+\varepsilon_{3} \end{array}$$

Where *α* is the intercept, *β* denotes the partial regression coefficients, *X* represents the independent variables, and *ε* is the residual error term not explained by the model.

### 2.5 Quality control

Before the formal survey, all research personnel underwent standardized training to ensure familiarity with the questionnaire content and mastery of appropriate survey techniques. A pilot study was conducted to test the survey procedures, identify potential issues, and refine the questionnaire and the overall research plan. Based on feedback, adjustments were made to ensure the reliability and validity of the survey instrument and methodology.

## 3 Results

### 3.1 Basic characteristics of the respondents

#### 3.1.1 Predisposing factors

A total of 511 older participants participated in the survey, comprising 154 males (30.14%) and 357 females (69.86%). The respondents ranged in age from 60 to 100 years, with the majority aged between 60 and 70 years(68.49%), followed by those aged 71–80 (18.20%), 81–90 (9.39%) and above 90 (3.91%). Regarding educational attainment, 261 respondents (51.08%) had completed junior high school or below, while 250 (48.92%) had received a high school education or higher, including college, university, and postgraduate degrees. In terms of marital status, 78.67% of the respondents were married or living with a spouse. Concerning the number of children, 98 individuals (19.18%) were childless; 161 (31.51%) had one child; 133 (26.03%) had two children; and 119 (23.29%) had three or more children. Regarding living arrangements, 80 respondents (15.66%) lived alone, 274 (53.62%) lived with a spouse, 150 (29.35%) lived with their children, and 7 individuals (1.37%) resided in nursing homes or other care facilities. As for occupational background prior to retirement, 100 respondents (19.57%) were public sector employees, 104 (20.35%) worked in enterprises, and 163 (31.90%) were farmers. In addition, 72 individuals (14.09%) were unemployed, while another 72 (14.09%) were engaged in various other occupations (see Table 2).

**Table 2:** Sample characteristics.

Variable	Category	*N*	Proportion (%)
Gender	Male	154	30.14%
	Female	357	69.86%
Age	60–70	350	68.49%
	71–80	93	18.20%
	81–90	48	9.39%
	>90	20	3.91%
Education	Junior high school and below	261	51.08%
	Senior school	100	19.57%
	Junior college	59	11.55%
	Bachelor degree	74	14.48%
	Master degree and above	17	3.33%
Marital status	No partner	109	21.33%
	Partnered	402	78.67%
Number of children	0	98	19.18%
	1	161	31.51%
	2	133	26.03%
	≥3	119	23.29%
Living arrangement	Alone	80	15.66%
	Nursing institution or other	7	1.37%
	Living with spouse	274	53.62%
	Living with children	150	29.35%
Pre-retirement occupation	Public institution personnel	100	19.57%
	Farmer	163	31.90%
	Other	72	14.09%
	Enterprise staff	104	20.35%
	No job	72	14.09%
Income per month (RMB)	3,000–5,000	149	29.16%
	<3,000	273	53.42%
	>5,000	89	17.42%
Source of Income	Other	61	11.94%
	Retirement pension	289	56.56%
	Business income	51	9.98%
	Government Aid	10	1.96%
	Children’s Provision	100	19.57%
Pension insurance	No	23	4.50%
	Yes	488	95.50%
Medical insurance	No	145	28.38%
	Yes	366	71.62%
Acceptable cost of medicine (RMB)	<1,000	352	68.88%
	1,000–2000	109	21.33%
	2000–3,000	33	6.46%
	≥3,000	17	3.33%
Convenience of community medical care	Inconvenience	48	9.39%
	Convenience	284	55.58%
	General	179	35.03%
Source of care at the time of consultation/hospitalization	Partner care only	98	19.18%
	One other source of care only	20	3.91%
	Child and child’s spouse care only	201	39.33%
	Two or more other sources of care	123	24.07%
	None	69	13.50%
Sources of financial help in case of medical consultation/hospitalization	Partner financial help only	70	13.70%
	One other kind of financial help only	29	5.68%
	Children and children’s spouses’ help only	186	36.40%
	Two and more kinds of other Financial help	145	28.38%
	None	81	15.85%
Children’s support	Do not support	101	19.77%
	Support	410	80.23%
Self-care ability	Need help from others	104	20.35%
	No self-care ability	6	1.17%
	Have self-care ability	401	78.47%
Have chronic disease	0	133	26.03%
	1	180	35.23%
	2	135	26.42%
	≥3	63	12.33%
Self-rated health	poor	52	10.18%
	Good	149	29.16%
	Fair	310	60.67%
Awareness of the CB-IHSC	Know well	28	5.48%
	Never heard	200	39.14%
	Heard but not understand	283	55.38%
Satisfaction with CB-IHSC	Comparatively satisfied	96	18.79%
	Not very satisfied	60	11.74%
	Very dissatisfied	28	5.48%
	Very satisfied	20	3.91%
	General	307	60.08%
Demand for the services	Yes	445	87.08%
	No	66	12.92%

Percentages are of the total sample; rounding may cause totals to differ from 100%.

#### 3.1.2 Enabling factors

Regarding monthly income, 29.16% of respondents reported earning between 3,000 and 5,000 RMB, 53.42% earning less than 3,000 RMB, and 17.42% earning more than 5,000 RMB. The primary source of income was retirement pensions (56.56%), followed by financial support from children (19.57%). Pension insurance coverage was high, with 95.50% of the respondents indicating they had it, and 71.62% of them also possessing medical insurance. Monthly medical expenses varied: 352 individuals (68.88%) spent less than 1,000 RMB, 109 (21.33%) spent between 1,000 and 2,000 RMB, 33 (6.46%) spent between 2,000 and 3,000 RMB, and 17 (3.33%) reported spending over 3,000 RMB. While the majority of respondents reported relatively easy access to medical care, 9.39% experienced difficulties in accessing treatment. In terms of care-giving support during outpatient visits or hospitalization, 201 individuals (39.33%) relied solely on their children or spouses; 123 (24.07%) had two or more sources of care; 98 (19.18%) were cared for only by a spouse; 20 (3.91%) relied on a single other caregiver, and 69 (13.50%) reported having no care-giving support. Regarding financial support for medical expenses, 186 respondents (36.40%) received assistance exclusively from their children or spouse; 145 (28.38%) had multiple financial support sources; 70 (13.70%) relied solely on their spouse; 29 (5.68%) relied on one other person, and 81 (15.85%) had no support from others in any form. When asked whether their children supported their participation in CB-IHSC, 410 individuals (80.23%) responded affirmatively, while 101 (19.77%) indicated a lack of support (see Table 2).

#### 3.1.3 Need factors

About the Self-care ability, 401 respondents (78.47%) were entirely self-sufficient, 104 (20.35%) partially dependent, and 6 (1.17%) entirely dependent. Concerning chronic disease status, most respondents reported having at least one chronic condition, while only 26.03% of them reported having no chronic illnesses. In terms of self-rated health status, 310 respondents (60.67%) considered their health to be good, 149 (29.16%) rated it as fair, and 52 (10.18%) assessed their health as poor. s of CB-IHSC was generally low: 283 individuals (55.38%) had heard of the model but lacked a clear understanding; 200 (39.14%) had never heard of it; and only 28 (5.48%) reported being very familiar with it. When asked about their need for CB-IHSC, 445 respondents (87.08%) expressed a clear demand, while 66 (12.92%) indicated no current need.

### 3.2 Analysis of factors influencing the demand for CB-IHSC with different characteristics

To identify variables significantly associated with the demand for CB-IHSC, bivariate analyses were performed using Chi-square tests for all predisposing, enabling, and need factors. As an example, Appendix Table A2 presents the cross-tabulation and Chi-square analysis between the convenience of community medical care and service demand. The analysis revealed a statistically significant association between these two variables (χ^2^ = 8.385, *p* = 0.015). The demand for CB-IHSC was highest (90.5%) among older adults who reported “Convenience” in accessing community medical care, followed by those reporting “General” convenience (87.3%). In contrast, the group reporting “Inconvenience” showed a comparatively lower demand rate (77.1%). This gradient suggests that better accessibility to local medical care is positively associated with a higher demand for integrated health and social care services. Following this approach, significant variables (*p* < 0.05) identified from the comprehensive bivariate screening are summarized in Table 1 below.

Among the predisposing factors, significant variables such as education, number of children, living arrangements, and occupation were found to have a statistically significant impact on the demand for CB-IHSC (*p* < 0.05). In terms of enabling factors, variables including income per month, acceptable cost of medicine, convenience of community care, sources of daily care and financial assistance during outpatient visits or hospitalization, as well as children’s support for CB-IHSC, were identified as significant factors (*p* < 0.05). For need factors, self-care ability, self-rated health, awareness of CB-IHSC were all statistically significant (*p* < 0.05), as seen in Table 1.

### 3.3 Logistic regression analysis

As demonstrated in Table 3, Model 3 (269.736) exhibited the lowest −2 log-likelihood value compared to Models 1 (348.491) and 2 (323.742). The smaller the −2 Log Likelihood, the better the model fit, indicating lower model error and better overall performance. The Cox & Snell R^2^ value for Model 3 was 0.215, which was higher than the values for Model 1 (0.084) and Model 2 (0.138). The Nagelkerke R^2^ value for Model 3 was 0.400, also surpassing that of Model 1 (0.156) and Model 2 (0.257). The Nagelkerke R^2^ increased from 0.156 (Model 1) to 0.257 (Model 2) and 0.400 (Model 3), indicating a progressive improvement in explanatory power as additional factors were included. The larger the Cox & Snell R^2^ and Nagelkerke R^2^ values, the better, as they represent a higher proportion of variance explained by the model. Model 3 has the lowest −2 Log Likelihood, and both the Cox & Snell R^2^ and Nagelkerke R^2^ are the highest, indicating the best fit. Additionally, from Model 1 to Model 2, the Nagelkerke R^2^increased by 0.101. From Model 2 to Model 3, the Nagelkerke R^2^ increased by 0.143, which is the largest improvement, confirming the most significant contribution of need-related variables and suggesting that these factors have the strongest influence in explaining the dependent variable. Therefore, Model 3 is the optimal model.

**Table 3:** Model fit statistics.

Model	−2 Log Likelihood	Cox & Snell R^2^	Nagelkerke R^2^
Model 1	348.491	0.084	0.156
Model 2	323.742	0.138	0.257
Model 3	269.736	0.215	0.400

As shown in Table 4, the *p*-values for Model 1 (*p* = 0.085), Model 2 (*p* = 0.135), and Model 3 (*p* = 0.413) were all greater than 0.05. Furthermore, the Hosmer-Lemeshow test also yielded p-values above 0.05, suggesting that the data extraction process was sufficient and that the model specification was appropriate. Among the models, Model 3 demonstrated the highest level of significance and the best overall fit. Therefore, Model 3 was selected for binary logistic regression analysis to examine how much each factor influences the demand for CB-IHSC among older participants.

**Table 4:** Hosmer–Lemeshow goodness-of-fit test.

Model	χ^2^	df	Sig.
Model1	13.893	8	0.085
Model2	12.377	8	0.135
Model3	15.988	8	0.413

Based on the results from Model 3, variables including the number of children, living arrangements, income per month, convenience of community care, support’s children, self-care ability, self-rated health, and awareness of the CB-IHSC were all found to be statistically significant (*p* < 0.05). These factors were identified as significantly impacting older participants’ demand for CB-IHSC. Detailed results are presented in Table 5.

**Table 5:** Logistic regression analysis including variables associated with CB-IHSC demand for the old adults.

Independent variable	B	*p*	OR	95% CI (Lower)	95% CI (Upper)
Education (Ref: Junior high and below)	0.090	0.608	1.09	0.78	1.55
Number of children (Ref: 0)	0.752	0.001	2.12	1.34	3.35
Living arrangement (Ref: Alone)	−0.741	0.011	0.48	0.27	0.85
Pre-retirement occupation (Ref: Public institution personnel)	−0.075	0.607	0.93	0.70	1.23
Income per month (RMB) (Ref: ><3,000)	−0.915	0.001	0.40	0.23	0.70
Acceptable cost of medicine (RMB) (Ref:<1,000)	−0.408	0.057	0.67	0.44	1.01
Convenience of community medical care (Ref: Inconvenience)	0.558	0.041	1.75	1.02	2.98
Source of care at the time of consultation/hospitalization (Ref: None)	0.073	0.611	1.08	0.81	1.42
Sources of financial help in case of medical consultation/hospitalization (Ref: None)	−0.145	0.290	0.87	0.66	1.13
Children’s support (Ref: Do not support)	1.310	0.003	3.71	1.55	8.85
Self-care ability	−1.745	0.001	0.18	0.08	0.38
Self-rated health (Ref: No self-care ability)	−1.089	0.002	0.34	0.17	0.67
Awareness of CB-IHSC (Ref: Never heard)	0.767	0.022	2.16	1.12	4.14
Satisfaction with CB-IHSC (Ref: Very dissatisfied)	0.013	0.948	1.01	0.68	1.51

OR, odds ratio; CI, confidence interval. Precision standardized (B: 3 decimals; OR/CI: 2 decimals; p: 3 decimals or “<0.001”).

## 4 Discussion

### 4.1 The demand for the CB-IHSC

The results of this study indicate a strong demand for CB-IHSC among older adults in Chengdu. Specifically, 87.08% of the respondents reported a need for such services, which is comparable to the demand level in Guangzhou (86.2%) (3) and slightly higher than that in Luzhou (66.3%) (2).

This elevated demand in Chengdu can be effectively interpreted through the Andersen Health Service Utilization Behavior Model. The city’s proactive policy support and investment in CB-IHSC complexes and creating a “15-min convenient living circle” have directly enhanced Enabling Resources by improving the accessibility and availability. Our findings confirm that convenient of community medical is a significant positive predictor of demand (OR = 1.747). Simultaneously, these government-led initiatives have shaped Predisposing Characteristics, particularly health beliefs, by raising public awareness and fostering a more positive attitude toward integrated care models. This is corroborated by our regression results, which identified awareness of CB-IHSC as a key driver of demand (OR = 2.155). Therefore, the high demand in Chengdu is not merely an external phenomenon but is likely mediated by the model’s core components: policy efforts improve enabling resources and shape predisposing characteristics, which in turn, as our model demonstrates, significantly influence the expressed demand for CB-IHSC.

### 4.2 Factors influencing demand

A key strength of this study is the hierarchical regression grounded in the Andersen Health Service Utilization Behavior Model, which allowed us to assess pre-specified hypotheses about the relative importance of factor domains. The results largely supported our hypotheses, yet several findings provided new insights. The data suggest that multiple factors—predisposing, enabling, and need factors—affect the demand for CB-IHSC among older adults in Chengdu. Specifically, the number of children and living arrangements were identified as significant predisposing factors; income, convenience of community medical care, and children’s support as enabling factors; and self-care ability, self-rated health, and awareness of CB-IHSC as key need factors. Each of these is discussed below.

#### 4.2.1 The composite role of children: quantity and support

Among the predisposing characteristics, the association between the number of children and higher demand for CB-IHSC (OR = 2.12) is one of our most surprising yet insightful findings. This is contrary to our H4 regarding the number of children. This positive relationship contrasts with the initial intuition that more children would substitute for formal services, as well as with the results of Xu Haijiao, who reported an inverse relationship between the number of children and demand for CB-IHSC suggesting that fewer children correlate with higher demand (17). Similarly, Li Qiang found that an increased number of children was associated with lower life satisfaction and higher levels of depression among their parents, indicating that more children do not necessarily bring more happiness (18). In families with multiple children, care responsibilities may be neglected or deferred, leaving older adults’ care needs unmet and prompting them to seek alternative models such as CB-IHSC.

As hypothesized in H4, regarding the support from children, it appears to be a significant positive factor influencing demand (OR = 3.71), aligning with the findings of Tan Mei and colleagues (19). When children express understanding and support for CB-IHSC, older adults’ confidence and demand for these services increase. Material and emotional support from children not only eases financial burdens but also reinforces the psychological readiness of older adults to adopt new care models.

This divergence finding underscores that the decision to utilize CB-IHSC is not solely determined by the structural aspect of family size (number of children), but is more significantly influenced by the functional aspect—the quality and nature of the support those children provide. A plausible explanation is that structural family size (quantity) does not translate into functional support unless accompanied by children’s active support (an enabling resource). Our analysis suggests the enabling dimension—children’s support for participation—shows a strong positive association with demand, indicating that who helps and how may matter more than how many children one has.

#### 4.2.2 Living arrangements

Living arrangements may influence the demand for the CB-IHSC with solitary living potentially linked to higher demand. Studies have shown that empty-nest older adults exhibit a hierarchical needs structure, prioritizing medical care, emotional support, and basic life assistance (20). Due to declining physical functions, older adults often require greater medical support and daily care. The CB-IHSC provide continuous support, such as health check-ups, rehabilitation, in-home care, and life assistance, which help meet these needs. Additionally, older adults living alone are more likely to experience loneliness and insecurity, increasing their reliance on CB-IHSC for social interaction and emotional support. Thus, as the number of cohabitants decreases, the demand for CB-IHSC tends to increase. This finding partially supports H5, which hypothesized that co-residence with family reduces demand due to informal care. However, the quality of family support is crucial; older individuals with limited or no caregiving capacity from family may still require formal care. Therefore, while co-residence often reduces demand, the need for CB-IHSC remains higher among those living alone, where informal care is insufficient.

#### 4.2.3 Monthly income

Contrary to hypothesis H3, monthly income was negatively associated with the demand for integrated care services. As income increases, the likelihood of choosing CB-IHSC decreases. This contradicts the findings of Nie Jie, who suggested that individuals with higher socioeconomic status were more likely to choose such services (21). However, the current results align with Zhi Mengjia and Xue Yuan, who found that higher-income individuals were more inclined to choose institutional care, while those with lower incomes preferred community or home-based care models (22, 23). This may be because traditional institutional care facilities have undergone years of development and offer better amenities and more comprehensive services, attracting wealthier older adults. In contrast, community-based care, which is more affordable, tends to appeal to those with lower incomes. The CB-IHSC is still relatively new and under development, so high-income individuals may remain cautious or hesitant to adopt it.

#### 4.2.4 Convenience of community medical care

As predicted by H2, the ease of accessing medical services within the community may influence demand, with more convenient access potentially linked to greater demand. This finding is consistent with the results of Lü Xinrui (24). When nearby medical institutions are available, frequent needs such as medication, follow-up visits, and basic nursing care can be addressed locally, thereby reducing travel time, financial costs, and physical risks (such as falls) associated with hospital visits. The convenient of community medical care enhances older adults’ trust in and reliance on CB-IHSC, thus increasing their willingness to use these services.

#### 4.2.5 Self-care ability and self-rated health

Self-care ability and health condition were strongly associated with demand for CB-IHSC. Poorer self-rated health may be associated with an increase in demand, consistent with H1. Physical function typically declines as people age, resulting in reduced self-care ability. Older adults with poorer health and lower levels of independence—particularly those who are semi-dependent or disabled—tend to have a higher demand for integrated care services compared to those who are self-sufficient. These findings are consistent with the study by Lin Qin (25). The CB-IHSC offers personalized care plans according to varying levels of dependency, covering both basic daily needs and professional medical care. This model remains applicable even as the health status of older adult’s changes, ensuring that their evolving care needs are effectively met.

#### 4.2.6 Awareness of the CB-IHSC

As expected and in support of H6, a positive correlation was observed between the level of awareness of the CB-IHSC and the demand for the services. Higher awareness may be linked to a greater intent to participate (26), aligning with the research of Zhang Yujie (27). In this study, only 28 respondents reported a comprehensive understanding of CB-IHSC; 283 had heard of it but did not understand it, and 200 reported never having heard of it. These findings indicate limited dissemination and awareness of CB-IHSC.

### 4.3 Limitations

This study has several limitations that should be considered when interpreting the results. First, the use of convenience sampling in main urban areas, while practical, means that our sample may not be fully representative of all older adults in Chengdu, particularly those in suburban or rural districts, or those who are completely home-bound and do not access community health services. Consequently, the applicability of our findings to the entire older population of Chengdu or other regions of China may be limited. Second, the cross-sectional design captures a snapshot in time and cannot establish causal relationships between the factors and service demand.

## 5 Conclusion and policy recommendations

This study identifies the factors influencing the demand for CB-IHSC among older adults in Chengdu. With the aging population and rising demand for CB-IHSC, it is critical to bridge gaps in service availability, accessibility, and awareness. Based on these findings, the following policy recommendations are proposed.

### 5.1 Improve service accessibility and quality

Efforts could be directed toward enhancing the accessibility and quality of CB-IHSC. Expanding and upgrading community-based facilities may contribute to equitable distribution and comprehensive coverage. CB-IHSC might be further developed by strengthening cooperation between community health centers and care institutions for older adults. Standardized protocols for service delivery, health management, and emergency response should be established to ensure continuity, safety, and effectiveness of care. Professional staff training could also be prioritized to improve service capacity.

### 5.2 Tailor services to individual needs

Given the varying health conditions, economic statuses, and family support structures of older adults, it is crucial to provide personalized and differentiated services. For instance, semi-dependent or disabled older adults may require more intensive medical and nursing support. At the same time, independent individuals may benefit more from health education, regular check-ups, and social activities. By implementing needs-based stratification and care planning, service efficiency and user satisfaction can be significantly improved.

### 5.3 Enhance family and social support systems

Family support remains a key enabling factor in older adults’ decisions to utilize integrated care services. Therefore, it is essential to strengthen the role of families in eldercare through policy incentives such as tax benefits, subsidies, or caregiver training programs. At the same time, community-based support networks could be developed to provide supplemental care and companionship, particularly for solitary or empty-nest older adults. Volunteer programs, inter-generational activities, and mutual-aid models may effectively complement formal care services.

### 5.4 Promote policy innovation and support

Local governments may continue to play a leading role by formulating long-term strategic plans and introducing targeted policies to support the development of CB-IHSC. It is crucial to increase financial investment, expand pilot programs, and disseminate best practices across communities. Furthermore, enhancing cross-sectoral coordination among departments such as health, civil affairs, and housing could support the sustainable development of these services. Additionally, promotional efforts should be enhanced using community bulletin boards, cultural events, and digital platforms (such as official WeChat accounts) to raise awareness and provide detailed information on available services. Increasing public understanding is essential to improving participation and ensuring that more older adults benefit from integrated care.

## Data Availability

The original contributions presented in the study are included in the article/Supplementary material, further inquiries can be directed to the corresponding author.
