# Supplementary material for: An analysis of factors influencing the demand for community-based integrated health and social care in Southwestern China
Source: Front Public Health. 2025 Nov 28;13:1684187. doi: 10.3389/fpubh.2025.1684187 (PMC12698466; doi:10.3389/fpubh.2025.1684187)
Supplement: Supplementary file 1 [file Table_1.docx]

**Appendix Table A1.** Variable definitions and coding (aligned with Table 2 & Table 5)

| **Block** | **Variable** | **Type** | **Level coding (verbatim)** |
| --- | --- | --- | --- |
| Outcome | Demand for CB-IHSC | Binary | 0 = No; 1 = Yes |
| Predisposing | Sex | Binary | 1 = Male; 2 = Female |
|  | Age group | Ordinal categorical | 1 = 60–70; 2 = 71–80; 3 = 81–90; 4 = >90 |
|  | Education | Ordinal categorical | 1 = Junior high school and below; 2 = High school; 3 = Junior college; 4 = Bachelor; 5 = Postgraduate |
|  | Marital status | Binary | 0 = No partner; 1 = Partnered |
|  | Number of children | Ordinal categorical | 1 = 0; 2 = 1; 3 = 2; 4 = ≥3 |
|  | Living arrangement | Nominal categorical | Alone; Living with spouse; Living with children; Nursing institution or other |
|  | Pre-retirement occupation | Nominal categorical | Public institution staff; Enterprise staffs; Unemployed; Farmer; Other |
| Enabling | Income per month | Ordinal categorical | 1 = <3000; 2 = 3000–5000; 3 = >5000 |
|  | Primary source of income | Nominal categorical | Pension; Children’s supply; Old-age insurance; Government assistance; Other |
|  | Medical insurance coverage | Binary | 0 = No; 1 = Yes |
|  | Old-age insurance coverage | Binary | 0 = No; 1 = Yes |
|  | Acceptable medical expenses | Ordinal categorical | 1 = ＜1000;2=1000-2000; 3 = 2000–3000; 4 = ≥3000 |
|  | Convenience of community medical care | Ordinal categorical | 1 = Inconvenience; 2 = General; 3 = Convenience |
|  | Source of care (during treatment/hospitalization) | Nominal categorical | None; Spouse only; Children & their spouses only; Other single source |
|  | Sources of financial help (during treatment/hospitalization) | Nominal categorical | None; Spouse only; Children & their spouses only; Other single source; Two or more other sources |
|  | Children’s support for participation in CB-IHSC | Binary | 0 = Not support; 1 = Support |
| Need | Self-care ability | Ordinal categorical | 1 = Completely dependent; 2 = Partially independent; 3 = Completely independent |
|  | Number of chronic conditions | Ordinal categorical | 1 = 0; 2 = 1; 3 = 2; 4 = ≥3 |
|  | Self-rated health (Health status) | Ordinal categorical | 1 = Poor; 2 = Fair; 3 = Good |
|  | Awareness of the CB-IHSC | Ordinal categorical | 1 = Never heard; 2 = Heard but not familiar; 3 = Very familiar |
|  | Satisfaction with the CB-IHSC | Ordinal categorical | 1 = Very dissatisfied; 2 = Somewhat dissatisfied; 3 = Neutral; 4 = Somewhat satisfied; 5 = Very satisfied |
|  | Choice of the CB-IHSC model | Binary | 0 = No; 1 = Yes |

**Notes.** Reference categories are listed explicitly in the last column and are consistent with the regression baselines reported in Table 5. Ordinal variables enter models as ordinal predictors with codes increasing from low to high; nominal variables use dummy coding with the listed Ref as baseline; binary variables use 0 = Ref and 1 = Other. Variable names are kept identical to those in Table 2.
