# Supplementary material for: An analysis of factors influencing the demand for community-based integrated health and social care in Southwestern China
Source: Front Public Health. 2025 Nov 28;13:1684187. doi: 10.3389/fpubh.2025.1684187 (PMC12698466; doi:10.3389/fpubh.2025.1684187)
Supplement: Supplementary file 2 [file Table_2.docx]

**Appendix Table A2.** Cross tab: Convenience of community medical care × Demand for CB‑IHSC (with Pearson Chi‑square)

| **Convenience of community medical care** | **No (count)** | **Yes (count)** | **Row % Yes** |
| --- | --- | --- | --- |
| Inconvenience | 11 | 37 | 77.1% |
| General | 28 | 151 | 84.4% |
| Convenience | 27 | 257 | 90.5% |
| Total | 66 | 445 | 87.1% |

**Pearson Chi‑square:** χ²(2) = 8.385, p = 0.015; **Cramér’s V:** 0.128; **N:** 511

**Notes.** Row percentages are within convenience categories. Counts are taken from the final Table 2; test based on 3×2 contingency table.
